# A Virus-Like Particle-Based Vaccine Candidate against the Tick-Borne Powassan Virus Induces Neutralizing Antibodies in a Mouse Model

**DOI:** 10.3390/pathogens10060680

**Published:** 2021-05-31

**Authors:** Velasco Cimica, Sahar Saleem, Emily Matuczinski, Debra Adams-Fish, Conor McMahon, Sujatha Rashid, Timothy T. Stedman

**Affiliations:** American Type Culture Collection (ATCC®), ATCC Federal Solutions, 10801 University Boulevard, Manassas, VA 20110, USA; ssaleem2@gmu.edu (S.S.); e.matuczinski@yahoo.com (E.M.); DAdams-Fish@atcc.org (D.A.-F.); cmcmahon@atcc.org (C.M.); srashid@atcc.org (S.R.); TStedman@atcc.org (T.T.S.)

**Keywords:** Powassan virus, vaccine, virus-like particle, POWV, VLP, flavivirus

## Abstract

Powassan virus (POWV) is a tick-borne flavivirus circulating in North America and the Russian Far East that can cause severe neuroinvasive diseases, including encephalitis, meningitis, and meningoencephalitis. The reported neuroinvasive case fatality is about 10%, and approximately 50% of the survivors from the neuroinfection exhibit long-lasting or permanent neurological sequelae. Currently, treatment of POWV infection is supportive, and no FDA-approved vaccines or specific therapeutics are available. A novel Powassan vaccine candidate was created using virus-like particle technology (POW-VLP) and assembled with the viral structural proteins pre-Membrane (prM) and Envelope (E). Western blot immunoassay demonstrated high antigenicity of POW-VLP structural proteins. Transmission electron microscopy indicated that the POW-VLP exhibited icosahedral morphology typical of flaviviruses. A dose-escalation study in a murine model was performed to test immunogenicity and safety. Serum antibody was tested by ELISA, demonstrating that POW-VLP afforded 100% seroconversion to the E protein. Reporter viral-particle neutralization assay demonstrated high levels of neutralizing antibodies in the serum of immunized mice. Hybridomas expressing monoclonal antibodies were produced following POW-VLP immunization. The POW-VLP vaccine candidate created in this study provides a strategy for inducing protective antibodies against Powassan neuroinvasive infection.

## 1. Introduction

Powassan virus (POWV) is a tick-borne flavivirus pathogen that was first discovered and isolated in Ontario, Canada, in 1958 [1]. POWV infections in humans are sporadic in North America and the Russian Far East [2,3,4]. The viral disease may initiate with flu-like symptoms, but about half of reported cases result in severe neuroinvasive disease, including encephalitis, meningitis, and meningoencephalitis. The fatality rate of neuroinvasive POWV cases is approximately 12%, and approximately 50% of neuroinvasive infections result in permanent brain damage associated with long-lasting neurological sequelae such as headaches, muscle weakness, focal paralysis, or cognitive difficulties [5]. Currently, treatment of POWV infection is supportive, and no FDA-approved vaccines or specific therapeutics are available.

In the USA, the incidence of POWV disease reported to the Centers for Disease Control and Prevention (CDC) has increased over the last two decades from about one case per year before 2005 to an average of more than 24 domestic cases annually since 2016 [6]. Importantly, a record of 39 cases was reported in 2019. Recent evidence suggests POWV is transmissible through blood transfusion, creating a risk for transmission through the blood supply [7]. POWV is characterized by two lineages serologically indistinguishable but circulating with a specific tick vector: lineage I is typically vectored by *Ixodes cookei*, while lineage II, also called deer tick virus (DTV), is usually transmitted by *Ixodes scapularis*. The emergence of POWV cases may have resulted from the virus lineage adaptation to the more aggressive tick species *Ix. scapularis* from the *Ix. cookei* that rarely bites humans. Indeed, *Ix. scapularis* has geographically expanded in recent decades across much of the eastern and central US [4] and is a primary vector for Lyme disease and the lineage II of POWV [8]. It is speculated that the combined effects of reforestation, urban sprawl, and greater human population density near the vector habitat [8] have driven increases in tick-borne diseases. Coincident with an increase in POWV infections, multiple states have reported their first confirmed cases in the last 5 years [9,10]. Given the increase in POWV incidence and expansion, human urbanization of rural areas, increase in the tick population, lack of therapeutics, and the potentially morbid consequences of POWV infection, proactive development of a POWV vaccine should be a public health priority.

Several VLP vaccine candidates have been developed employing the E protein as antigen [11,12]. Flavivirus E protein comprises the majority of the virion structure and surface [13]. The E protein plays multiple roles in the viral infection cycle, including host receptor recognition and binding, membrane fusion with the cell, virion maturation, assembly, and egress from the cell [14]. Indeed, most flavivirus vaccines deliver the E protein as an immunogen due to the induction of virus-neutralizing and protective antibodies.

The flavivirus E protein has a conserved structure divided into three domains. These include the amino-terminal domain I (EDI) consisting of a beta-barrel within the middle of the protein monomer; domain II (EDII), which includes a fusion peptide that is functionally important for dimerization and virion assembly; and the carboxy-terminal domain III (EDIII), which is characterized by an immunoglobulin-like domain. Multiple studies have demonstrated that EDIII is the ideal antigen for eliciting neutralizing and protective antibodies against both tick-borne [15,16] and mosquito-borne flaviviruses [17,18].

Herein, we report the development of the first virus-like particle (VLP)-based vaccine candidate against POWV. VLP vaccines are based on the self-assembly of viral proteins into a structure resembling the native virion. The morphology of VLPs enhances epitope presentation and immune stimulation for both humoral and cellular responses [19]. VLPs exhibit high safety profiles because they lack the genetic material required for viral replication [20]. We produced Powassan VLPs (POW-VLP) in mammalian cells using the prM-E structural polyprotein. The POW-VLP exhibited the structural morphology of icosahedral virions. The immunization of mice with POW-VLPs induced neutralizing antibodies, and monoclonal antibodies targeted to POWV E protein were generated.

## 2. Results

### 2.1. Characterization of POWV EDIII Recombinant Protein

**Figure 1:**
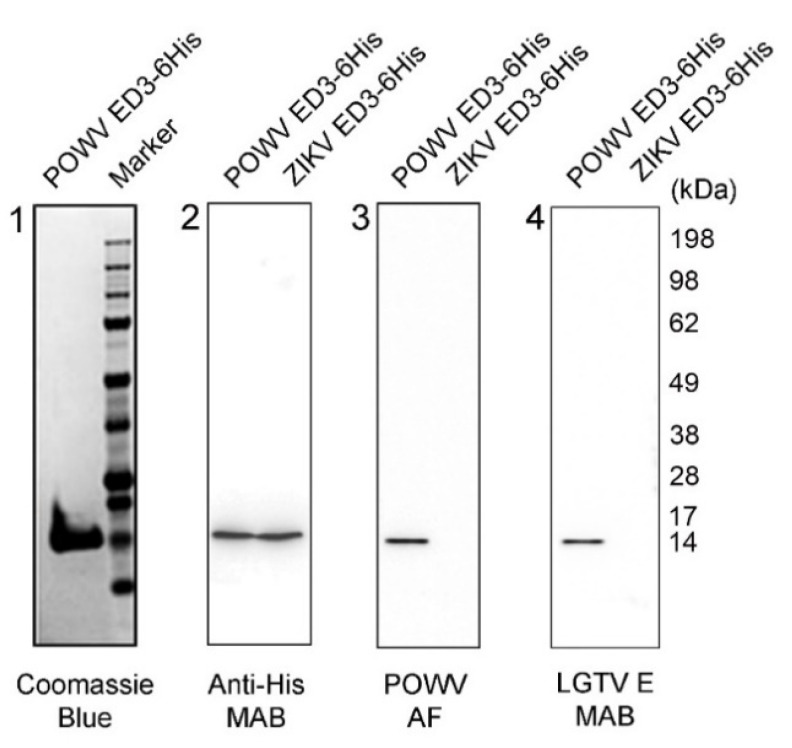
Analysis of POWV EDIII recombinant protein by SDS-PAGE and Western blot. Coomassie blue staining indicated electrophoretic mobility of approximately 12.7 kDa (left panel 1). Western blot analysis of POWV EDIII and ZIKV EDIII control (panels 2 to 4 from left). The anti-histidine monoclonal antibody (Anti-His MAB) detected both recombinant proteins (panel 2). Anti-POWV ascitic fluid antibody (POWV AF) (panel 3) and LGTV anti-envelope protein monoclonal antibody (LGTV E MAB) (panel 4) reacted specifically with POWV EDIII and not with ZIKV EDIII.

A truncated POWV E protein encompassing domain III (EDIII) was engineered as a control antigen. The POWV EDIII was expressed with a C-terminal histidine tag for detection and purification by immobilized metal affinity chromatography (IMAC). The recombinant protein was produced in a yeast system (*Pichia pastoris*) with a high yield of expression. SDS-PAGE and Coomassie blue staining analysis showed over 95% purity of POWV EDIII (Figure 1, panel 1), with electrophoretic mobility in agreement with the predicted molecular weight of 12.7 kDa obtained by bioinformatics analysis [21]. Western blotting analysis showed POWV EDIII reactivity using anti-histidine antibody and aligning with a similar molecular size and reactivity with respect to a Zika Virus (ZIKV) EDIII histidine-tag recombinant protein standard (Figure 1, panel 2). In addition, POWV EDIII reacted specifically with anti-POWV ascitic fluid antibody (POWV AF) (Figure 1, panel 3). The monoclonal antibody against E protein from the closely related Langat virus (LGTV) that shares the same serocomplex with POWV [22] was cross-reactive with the POWV EDIII recombinant protein (Figure 1, panel 4). Neither POWV AF nor LGTV E antibodies cross-reacted with the more divergent ZIKV EDIII recombinant protein (Figure 1, panels 3, 4).

### 2.2. Production of POW-VLPs in a Mammalian System

**Figure 2:**
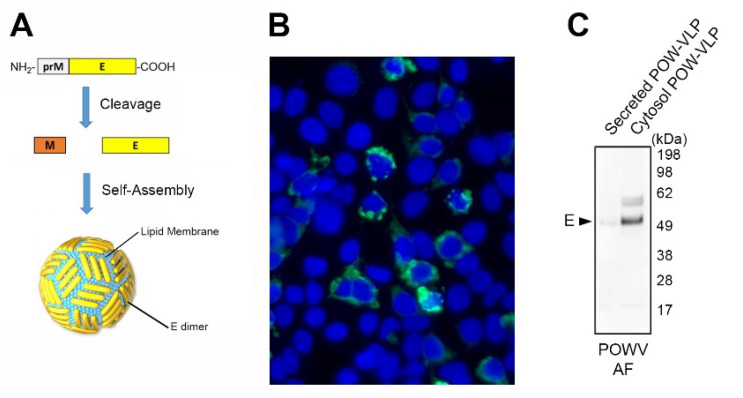
Production of POW-VLP in mammalian cells. (**A**) The expressed recombinant E protein used for POW-VLP generation undergoes processing and self-assembly into virus-like particles. (**B**) Indirect immunofluorescence detection of the POWV E protein expressed in transfected mammalian cells. (**C**) Purified POW-VLPs were detected by Western blot analysis from cell culture supernatants and the cytosolic extract of transfected mammalian cells. POW-VLPs were poorly secreted and were primarily retained in the cytosol. Both assays were performed using anti-POWV ascitic fluid antibodies.

An expression construct encoding the polyprotein truncation prM-E was engineered for the expression of POW-VLP in mammalian cells (Figure 2a). The POW-VLP construct was transfected into 293T cells, and POWV E expression was confirmed by indirect immunofluorescence with a POWV ascitic fluid antibody. POWV E protein was abundant in the extranuclear compartment of the transfected cells as expected (Figure 2b). Furthermore, Western blot analysis demonstrated that POW-VLP was poorly secreted in the culture media and accumulated in the cytosolic fraction (Figure 2c). The bulk of the POWV E protein migrated to approximately 54 kDa as predicted for the mature protein in agreement with the literature and bioinformatics analysis [21,23], with a minor band present at an apparent molecular weight of approximately 60 kDa, which may represent the uncleaved M-E protein.

### 2.3. POW-VLPs Demonstrate High Antigenicity and Virion Morphology

**Figure 3:**
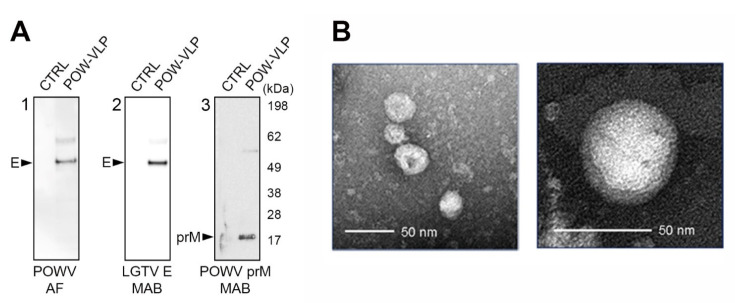
Characterization of POW-VLPs. (**A**) POW-VLP antigenicity assessed by Western blot analysis. Anti-POWV ascitic fluid antibodies (POWV AF) (panel 1) and LGTV anti-envelope protein monoclonal antibodies (LGTV E MAB) (panel 2) recognized E protein from POW-VLP; the presence of prM protein from POW-VLPs was detected using POWV anti-prM antibody (panel 3). (**B**) Transmission electron microscopy analysis indicated icosahedral morphology and size of 40–50 nm for POW-VLP.

Purified POW-VLP were characterized for antigenicity using multiple monoclonal antibodies. POWV E protein was detected with both POWV ascitic fluid and LGTV anti-E protein monoclonal antibodies (Figure 3A). Both antibodies detected a major band with a molecular weight of approximately 54 kDa (Figure 3A, panels 1 and 2). The prM protein was detected with another monoclonal antibody presenting a band with a molecular size of approximately 18 kDa (Figure 3A, panel 3). Virion morphology of POW-VLPs was examined by transmission electron microscopy (TEM) and negative staining. The VLPs exhibited icosahedral morphology typical of flavivirus and sizes between 40 and 50 nm, in agreement with previous studies regarding POWV [24,25].

### 2.4. POW-VLPs Elicit Antibody Response against POWV EDIII Protein

POW-VLPs were produced and purified at a large scale in mammalian cells as described above. The concentration of POW-VLP was measured by dot blot immunoassay using a POWV ascitic fluid antibody and POWV-EDII recombinant protein as a molecular standard. The POW-VLP immunization dose was based on the quantity of EDIII antigen delivered. 

A dose-escalation study for POW-VLP was performed in BALB/c mice. The immunization of 10 mice per group (5 male, 5 female) was completed by intramuscular injection using a prime and 2 boosts immunizations regimen separated by 3-week intervals. Mice were dosed with 1, 5, or 10 µg of POW-VLP containing EDIII equivalent antigen. In order to increase vaccine efficacy, POW-VLPs were formulated with an adjuvant composed of a squalene-based oil-in-water nano-emulsion (AddaVax).

Mice were observed daily during the entire study, and no adverse effects were found from the immunization protocol, demonstrating the vaccine tolerability.

**Figure 4:**
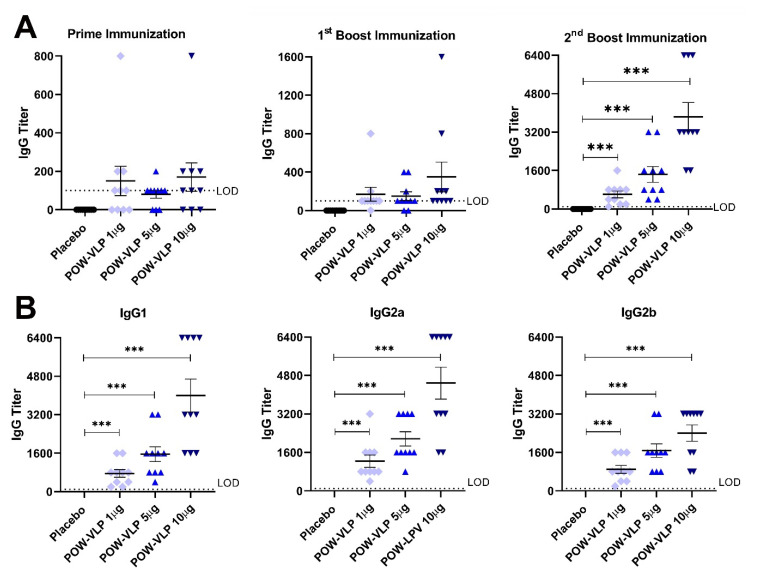
Testing of POW-VLP immunogenicity by ELISA. (**A**) POW-VLPs induced IgG against POWV EDIII antigen after prime immunization. IgG titer increased after boost immunizations, showing 100% seroconversion in each dosage group (*** *p* < 0.0005). (**B**) POW-VLP elicited both IgG_1_ and IgG_2_ (**A**,**B**) antibody response after second boost immunization (*** *p* < 0.0005).

Serum samples were collected 3 weeks after prime, first boost, and second boost immunizations and analyzed by ELISA with POWV EDIII recombinant protein as a coating antigen (Figure 4A). The prime immunization resulted in roughly 70% seroconversion in the immunized mice with no statistical difference in the serum titer between groups (Figure 4A). The serum titer increased after the first boost immunization, reaching a complete seroconversion for the highest dose of 10 µg of POW-VLP. The second boost immunization further augmented the antibody response and elicited complete seroconversion from the lowest to the highest dose of POW-VLP. After the final immunization, the serum antibody response was found to be dose-dependent. 

The IgG isotype antibody response was analyzed by ELISA of serum samples collected after the final immunization (second boost) (Figure 4B). POW-VLPs elicited both IgG_1_ and IgG_2_ (a and b), corresponding to Th2- and Th1-immune responses, respectively.

### 2.5. POW-VLP Induces Neutralizing Antibodies

**Figure 5:**
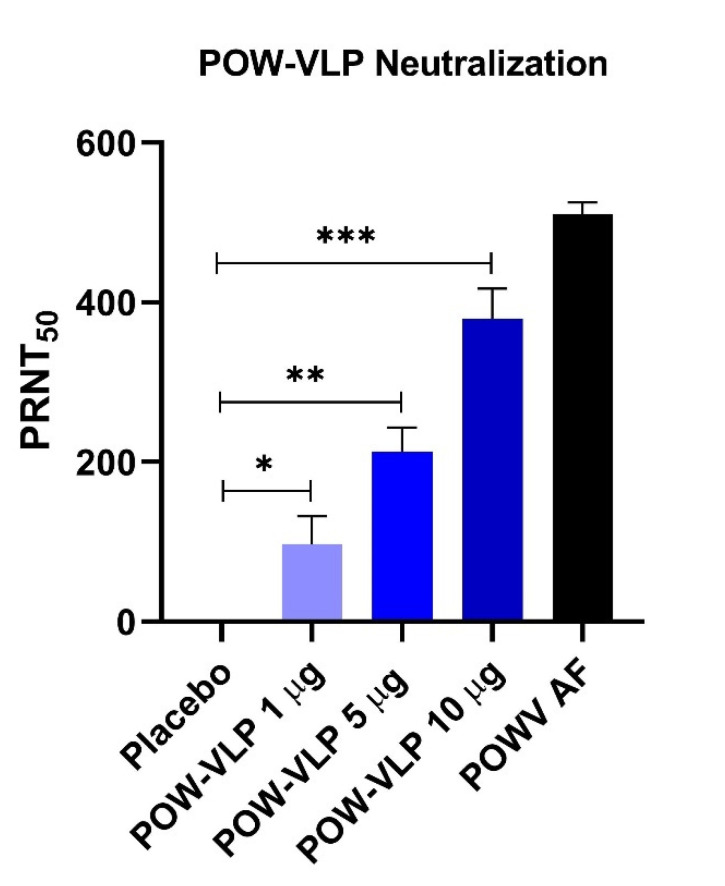
Neutralization test for POW-VLP immunization in a murine model. The reporter virus-particle neutralization test demonstrated that POW-VLPs induce a dose-dependent neutralization activity in a murine model (* *p* < 0.05; ** *p* < 0.005; *** *p* < 0.0005). Neutralization activity quantification is indicated by 50% inhibition of plaque reduction neutralization test (PRNT_50_). Anti-POWV ascitic fluid antibody was included as a control (POWV AF).

Pooled serum samples collected 3 weeks after the second boost immunization of POW-VLPs were analyzed for neutralization activity using a reporter virus-like particle neutralization test established by Pierson et al. [26]. POW-VLPs elicited a high level of neutralizing antibodies in a dose-dependent manner for each of the 3 dosages of the POW-VLP vaccine (1, 5, and 10 µg of EDIII protein equivalent antigen) (Figure 5). Each dosage group demonstrated statistically significant neutralization activity with respect to placebo control. The high dose of 10 µg of POW-VLP elicited antibodies with neutralization activity comparable to anti-POWV ascitic fluid (AF) antibody-positive control.

### 2.6. Generation of Monoclonal Antibodies against POWV 

**Figure 6:**
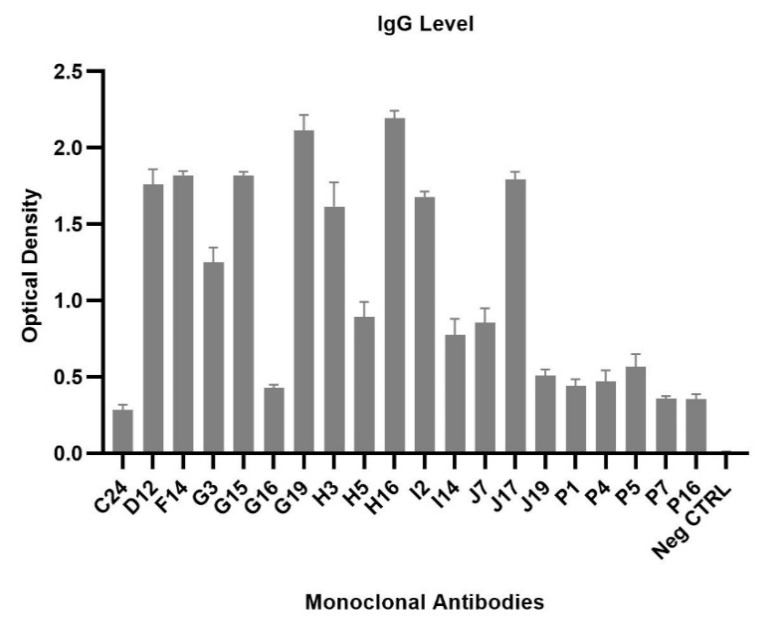
ELISA screening for monoclonal antibodies. Twenty hybridomas expressing monoclonal antibodies were screened for secretion of IgG against POW-EDIII recombinant protein antigen. Optical density values were subtracted against the background control value.

Splenocytes were harvested from a mouse immunized with a high dosage of POW-VLP (10 µg EDIII equivalent antigen), and hybridoma clones were isolated for monoclonal antibody production. Hybridoma clones were screened by ELISA using POWV EDIII as a coating antigen. Twenty positive clones were assessed for reactivity by ELISA screening (Figure 6).

**Figure 7:**
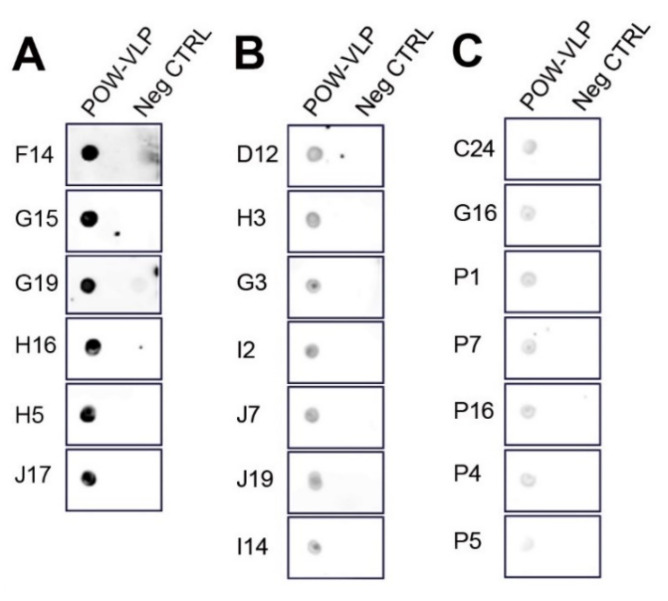
Hybridoma testing by dot blot immunoassay. Supernatant from the hybridoma culture was tested for antibody reactivity against the POW-VLP antigen. Hybridomas were classified as (**A**) high, (**B**) medium, and (**C**) low reactivity. Protein lysate was used as a negative control.

Hybridoma clones with varying levels of reactivity to POW-EDIII were selected for further screening. Hybridoma supernatants were tested against the POW-VLP antigen by dot blot immunoassay (Figure 7). The dot blot analysis correlated with the ELISA screening showed above (Figure 6 and Figure 7), demonstrating monoclonals with high (Figure 7A), medium (Figure 7B), and low reactivity (Figure 7C). 

## 3. Discussion

Due to the success of the VLP platform, multiple flavivirus vaccine candidates have been developed. The ability of flavivirus prM-E or C-prM-E polyprotein to self-assemble into an enveloped VLP with similar morphology to the wild-type virus has been harnessed to create highly immunogenic recombinant vaccines [27,28]. 

We have developed a novel VLP-based vaccine candidate against the tick-borne POWV. To date, only two nucleic acid-based vaccine candidates have been developed against Powassan infection, each exhibiting some efficacy and safety [29,30]. However, RNA-based vaccines require stringent cold chain maintenance for storage and distribution, whereas VLPs are quite stable under standard refrigeration, potentially facilitating the distribution. A VLP-based vaccine presents conformational epitopes from the virion structure that stimulate highly neutralizing and protective antibodies [19,20].

The prM-E based VLPs are efficiently secreted from mammalian and insect cells into the culture media, making this approach convenient for the manufacturing process. While the POW-VLP vaccine candidate was produced robustly in mammalian cells using virus prM-E structural proteins, the expressed POW-VLPs remained predominantly in the intracellular compartment, with low secretion into the culture supernatant. The low degree of secretion suggests that POWV requires the capsid (C) and non-structural (NS) proteins for complete virion maturation, efficient budding, and release from the cell [31,32]. 

Two recent reports demonstrated that the EDIII antigen from POWV or TBEV elicits broadly neutralizing and protective antibodies against tick-borne flaviviruses. Monoclonals antibodies developed against POWV EDII or EDIII inhibited post-attachment viral entry in the cell [33]. A subset of cross-reacting monoclonal antibodies protected against POWV, Langat virus (LGTV), and TBEV infection in a mouse model. In another study, broadly neutralizing antibodies against tick-borne flavivirus were discovered in humans after TBEV infection [34]. The antibodies were able to bind the TBEV EDIII domain and exert broad neutralization activity against multiple tick-borne flaviviruses, including POWV. One isolated monoclonal antibody demonstrated high protection against TBEV infection in a mouse model. 

The POW-VLP vaccine candidate produced in this study exhibited high antigenicity and elicited serogroup-specific antibodies against POWV EDIII (Figure 1 and Figure 3A). Using ELISA, we measured the serum antibody response specific to EDIII induced by POW-VLP. However, since POW-VLP expresses the full E protein and epitopes spanning EDI, EDII, prM, and VLP structural antigens, the current analyses may underestimate the total IgG titer against the virus. Hence, the POW-VLP vaccine candidate may induce additional protective antibodies from other epitopes.

The structural assembly of the VLP ensures the conformationally correct presentation of EDIII epitopes to the host immune system. The external shell of a mature flavivirus virion is composed of 180 copies of the E protein arranged in head-to-tail dimers organized on the icosahedral surface of the virion [35]. Transmission electron microscopy analysis demonstrated that POW-VLPs are assembled with icosahedral morphology and size comparable to the native virus (Figure 3B) [24]. 

*In vivo* studies demonstrated the POW-VLP stimulated neutralizing antibodies specific for the EDIII domain (Figure 4 and Figure 5). We have used these preliminary studies to produce monoclonal antibodies against POWV. We isolated 20 hybridoma clones producing E protein-specific antibodies reactive against both POWV EDIII protein and POW-VLPs (Figure 6 and Figure 7). Such monoclonal antibodies represent potential candidates for therapeutic intervention against POWV infection. In conclusion, our study describes a novel POW-VLP vaccine candidate able to induce antibodies against POWV, which correlated with high neutralization activity. This outcome holds potential for the development of a safe and efficacious VLP-based POWV vaccine.

## 4. Materials and Methods

### 4.1. Generation of POWV EDIII Recombinant Protein

A recombinant form of the truncated Envelope Domain III (EDIII) protein of the POWV LB strain (GenPept: NP_775516) was produced by the *Komagataella phaffii* (*Pichia pastoris*) expression system and purified using nickel affinity chromatography. POWV EDIII contains residues 301 to 401 of the EDIII protein and features a thrombin cleavage site and C-terminal octa-histidine tag (BEI Resources, Manassas, VA, USA) (Cat. NR-52391).

### 4.2. Production of POW-VLPs

A plasmid construct expressing POW-VLP was designed by ATCC and was synthesized by ATUM (Newark, CA, USA). The backbone vector provided the CMV transcription promoter (Cat. pD2529-CMV-03), and the gene cassette was designed to express the prM-E gene from the POWV LB strain (GenBank NC_003687, amino acid 111–775) [36]. The POW-VLP were produced by transfection of a prM-E construct in an early passage cell culture of 293T (ATCC^®^, Manassas, VA, USA) (Cat. CRL-3216™) using Lipofectamine 3000 transfection reagent (Thermo Fisher Scientific, Waltham, MA, USA) (Cat. L3000001) following the manufacturer’s instructions. The culture media consisted of DMEM culture media (ATCC^®^, Manassas, VA, USA) (Cat. 30-2002™) supplemented with 10% fetal bovine serum (FBS) (ATCC^®^, Cat. 30-2020^™^). After 24 h of the transfection, the culture media was replaced. Cells were harvested 72 h after transfection using a mammalian cell lysis kit (Sigma-Aldrich, St. Louis, MO, USA) (Cat. MCL-1) following the manufacturer’s instructions and omitting SDS in the lysis buffer. Cell lysates were clarified by centrifugation for 30 min at 10,000× *g*. VLPs were concentrated by ultracentrifugation for 2 h at 140,000× *g* with a 20% sucrose cushion in phosphate-buffered saline (PBS). Pellets were resuspended in PBS. VLPs were subsequently purified by ultracentrifugation through a continuous sucrose gradient (10% e 50%) for 4 h at 180,000× *g*. VLPs were collected from the 20% and 30% fractions and further purified by ultrafiltration using Amicon ultra-15 filter units with a 100 kDa cutoff (Millipore, Burlington, MA, USA) (Cat. UFC910008). Cell cultures were incubated at 37 °C, 95% humidity, and 5% CO_2_ in all studies.

### 4.3. Immunofluorescence Method 

VLP production in mammalian cell culture was detected by immunofluorescence assay (IFA) on the transfected cells. Cells, 293T (ATCC^®^, Manassas, VA, USA) (Cat. CRL-3216™), were plated in chamber slides (Thermo Fisher Scientific, Waltham, MA, USA) (Nunc Lab-Tek Chamber Slide) and, after 24 h, were transfected with POW-VLP construct as described above. A total of 48 h after the transfection, the cells were fixed with ice-cold methanol for 10 min and washed thrice with PBS. The slides were incubated for 1 h with a blocking buffer (3% non-fat dry milk in PBS). After blocking, the slides were washed thrice with PBS and incubated with antibody Powassan virus immune ascitic fluid [V-518-711-562] (ATCC^®^, Cat. VR-1262AF™), diluted to 1:100 for 1 h. The slides were washed thrice and incubated for 1 h with goat anti-mouse IgG antibody, FITC conjugate (Millipore, Burlington, MA, USA) (Cat. 5008). After washing the slides thrice with PBS, Vectashield mounting media containing DAPI for nuclear staining (Vector Laboratories, Burlingame, CA, USA) (Cat. H-1200-10) was used for preparing the slides for the epifluorescence microscopy. Cell cultures were incubated at 37 °C, 95% humidity, and 5% CO_2_ in all studies.

### 4.4. Dot Blot 

Antigen concentration of the POW-VLP vaccine was assayed using dot blot analysis as described before [37]. Using a volumetric range of 1 to 5 μL, VLP samples were absorbed on nitrocellulose for 15 min and blocked for 1 h with a blocking buffer consisting of 3% non-fat dry milk in PBS containing 0.05% Tween 20 (PBS-Tween). A standard curve was obtained using recombinant Powassan virus LB EDIII protein containing a C-terminal histidine tag (BEI Resources, Manassas, VA, USA) (Cat. NR-52391). The dot blot membrane was incubated for 2 h with primary antibody Powassan virus immune ascitic fluid [V-518-711-562] (ATCC^®^, Manassas, VA, USA) (Cat. VR-1262AF™), diluted in blocking buffer 1:250. After washing in PBS-tween, the membrane was incubated for 1 h with secondary antibody solution, goat anti-mouse IgG antibody, HRP Conjugate (ImmunoReagents, Raleigh, NC, USA) (Cat. GtxMu-003-DHRPX) diluted in blocking buffer 1:5000. After a final washing with PBS-tween, the detection was performed using enhanced chemiluminescence (ECL) substrate Radiance Q (Azure Biosystems, Dublin, CA, USA) and a digital imaging system (Azure Biosystems c600). Antigen quantification was performed using ImageJ imaging software (NIH, Bethesda, MD, USA). 

### 4.5. Western Blot

Western blot was performed using Bolt 4–12% Bis-Tris Plus gels, 10-wells (Thermo Fisher Scientific, Waltham, MA, USA) (NW04120BOX) following the manufacturer’s instructions. Primary and secondary antibodies used for the immunoassays included Powassan virus immune ascitic fluid [V-518-711-562] (ATCC^®^, Manassas, VA, USA) (Cat. VR-1262AF™), monoclonal anti-Langat virus envelope glycoprotein (E), clone 10F6 (BEI Resources, Manassas, VA, USA) (Cat. NR-40316), Powassan virus prM protein antibody (GeneTex, Irvine, CA, USA) (Cat. GTX132055), His-Tag antibody (H-3) (Santa Cruz Biotechnology, Dallas, TX, USA) (Cat. sc-8036), goat anti-mouse IgG antibody, and HRP conjugate (ImmunoReagents, Raleigh, NC, USA) (Cat. GtxMu-003-DHRPX). Detection was performed using enhanced chemiluminescence (ECL) substrate Radiance Q (Azure Biosystems, Dublin, CA, USA) and a digital imaging system (Azure Biosystems c600). Recombinant protein Zika Virus EDIII His-Tag was used as a protein standard (Sino Biological US, Wayne, PA, USA) (Cat. 40543-V08Y). 

### 4.6. Electron Microscopy

VLP preparations were negatively stained and examined by transmission electron microscopy (TEM) at the George Washington University Nanofabrication Imaging Center, Washington, DC. Suspended particles (10 μL) were applied to a glow discharged Formvar carbon-coated copper grid and incubated for 1 min. The VLPs were fixed on grids with 4% glutaraldehyde for 5 min followed by water washes. The samples were then stained with 2% aqueous uranyl acetate for 1 min. The grids were examined at 80 kV using an FEI Talos F200X TEM (Thermo Fisher Scientific, Waltham, MA, USA).

### 4.7. Murine Model for POW-VLP Immunization

BALB/c mice (*Mus musculus*), 6-week-old males and females (Envigo, Frederick, MD, USA) were immunized by intramuscular (i.m.) injections of 50 μL of POW-VLP vaccine or a placebo control. Each experimental group consisted of 5 females and 5 males. Immunizations were administered on days 1, 21 and 42, and each VLP vaccine dose contained POW-VLPs admixed in a 1:1 volume with AddaVax, a squalene-based oil-in-water nano-emulsion (InvivoGen, San Diego, CA, USA) (Cat. vac-adx-10). The placebo group received PBS admixed with AddaVax at a 1:1 volume. Serum was collected by tail vein bleeding before and after immunizations and retro-orbital bleeding at the end of the study.

### 4.8. ELISA

Serum antibody response to POW-VLP immunization was analyzed by direct ELISA, using individual serum samples. Each well of the ELISA plates Costar (Corning Inc., Corning, NY, USA) was coated with 25 ng of recombinant Envelope Domain III (EDIII) protein from the Powassan virus (BEI Resources, Manassas, VA, USA) (Cat. NR-52391) diluted in PBS and incubated at 4 °C overnight. Serum samples were serially diluted in blocking buffer (3% non-fat dry milk in PBS-Tween), applied in triplicate to the coated ELISA plates, and incubated for 2 h at room temperature. Following washes with PBS-Tween (PBS plus 0.05% Tween-20), the detection was carried out using HRP conjugated anti-mouse antibodies recognizing IgG or IgG subtypes (Bio-Rad Laboratories, Hercules, CA, USA), diluted 1:10,000 in a blocking buffer, and applied to the ELISA plates for 1 h. After a final washing with PBS-tween, the detection was performed using enhanced chemiluminescence (ECL) substrate Radiance Q (Azure Biosystems, Dublin, CA, USA). and a microplate reader with a luminescence detection system EnVision (PerkinElmer, Waltham, MA, USA). Antibody titer was calculated using a serial dilution of serum, and the endpoint titer was defined as the reciprocal of the highest serum dilution that gives a reading above the cutoff value [38]. The cutoff value was calculated as follows: cutoff = mean negative control + (3 times standard deviation) [39].

### 4.9. Neutralization Assay

The neutralization assay was performed using the reporter viral particle system established by the Pierson laboratory [26] and plaque reduction neutralization test (PRNT). An early passage cell culture of 293T (ATCC^®^, Manassas, VA, USA) (Cat. CRL-3216™) was plated into a 6 well-plate using about 0.3 × 10^6^ cells in 2 mL of DMEM culture media (ATCC^®^, Cat. 30-2002™) supplemented with 10% fetal bovine serum (FBS) (ATCC^®^, Cat. 30-2020™), a day before transfection. Transfection was performed using 500 μL of OptiMEM (Thermo Fisher Scientific, Waltham, MA, USA), 1 μg of replicon plasmid pWNVII-Rep-G-Z, 3 μg of packaging plasmid POWV C-prM-E, and transfection reagent Lipofectamine 3000 (Thermo Fisher Scientific, Waltham, MA, USA) (Cat. L3000001) following the manufacturer’s instructions. The transfection reaction was incubated for 30 min at room temperature and added to the culture dropwise. The cell media was changed after 24 h, and reporter viral particles were harvested 2 days after transfection. Titration of reporter viral particles was achieved by inoculation to a confluent monolayer of Vero cells (ATCC^®^, Cat. CCL-81™) growing in 24 well-plate using EMEM culture media (ATCC^®^, Cat. 30-2003™) supplemented with 2% FBS (ATCC^®^, Cat. 30-2020™). Green fluorescence protein (GFP) foci were counted using a fluorescence microscope 2–3 days following inoculation. Neutralization activity was tested by mixing a constant amount of reporter viral particles and serial dilutions of immunized mouse serum. The neutralization reactions were incubated for 1 h at 37 °C before inoculation to Vero cells. The 50% inhibition by the plaque reduction neutralization test (PRNT_50_) was calculated by dose-response curve analysis GraphPad Prism 8 (GraphPad Software, San Diego, CA, USA). Cell cultures were incubated at 37 °C, 95% humidity, and 5% CO_2_ in all studies.

### 4.10. Generation of Hybridomas Expressing Monoclonal Antibodies

Hybridomas were established using a spleen from a mouse immunized with a high dose of POW-VLP (10 µg) following the vaccination protocol described above. The hybridoma generation was obtained using the ClonaCell-HY hybridoma kit (Stemcell Technologies, Vancouver, Canada) (Cat. #03800) following the manufacturer’s instructions and using a semi-solid medium for the initial selection of clones. Hybridoma culture supernatant secreting antibodies were screened by ELISA using POWV EDIII recombinant protein as a coating antigen as described above. Dot blot analysis was employed for testing antibodies in hybridoma supernatants, using 500 ng of POW-VLP and equal amounts of protein from cell lysate as a negative control, using a protocol described above. Cell cultures were incubated at 37 °C, 95% humidity, and 5% CO_2_ in all studies.

### 4.11. Statistics

Data were statistically analyzed and graphed using GraphPad Prism 8 (GraphPad Software, San Diego, CA, USA). Statistical significance was measured using Student’s *t*-tests between experimental groups. Images were processed using Adobe Photoshop (Adobe).

### 4.12. Ethics Statement

The animal study was conducted according to the ARRIVE guidelines (https://arriveguidelines.org/ accessed on 10 August 2018), and approved by the Institutional Review Board (see below). This manuscript follows ICMJE (http://www.icmje.org/ accessed on 30 December 2019) and journal guidelines regarding conflict of interest and disclosures. 

## 5. Conclusions

In summary, we describe the creation of a novel VLP-based vaccine candidate against POWV displaying the EDIII epitope for elicitation of highly neutralizing antibodies *in vivo*. This study demonstrated that POW-VLP stimulated the production of serum neutralizing antibodies that correlate with the development of strong Th1- and Th2-mediated immune responses. Furthermore, immunization with this vaccine candidate induced the generation of monoclonal antibodies against POWV EDIII protein. We anticipate that the POW-VLP vaccine will induce broad-spectrum neutralizing antibodies against EDIII within the natural virion structure and thus afford protection against Powassan neuroinvasive infection. Based on preliminary assessment in the murine model, POW-VLP represents a viable, safe, and efficacious vaccine candidate for challenge studies and preclinical evaluation.

## Data Availability

The data presented in this study has been included in the manuscript.
